# Supplementary material for: Molecular Identification of Pro-Excitogenic Receptor and Channel Phenotypes of the Deafferented Lumbar Motoneurons in the Early Phase after SCT in Rats
Source: Int J Mol Sci. 2022 Sep 22;23(19):11133. doi: 10.3390/ijms231911133 (PMC9569670; doi:10.3390/ijms231911133)
Supplement: Supplementary file 1 [file ijms-23-11133-s001.zip › ijms-1866093-supplementary.pdf]

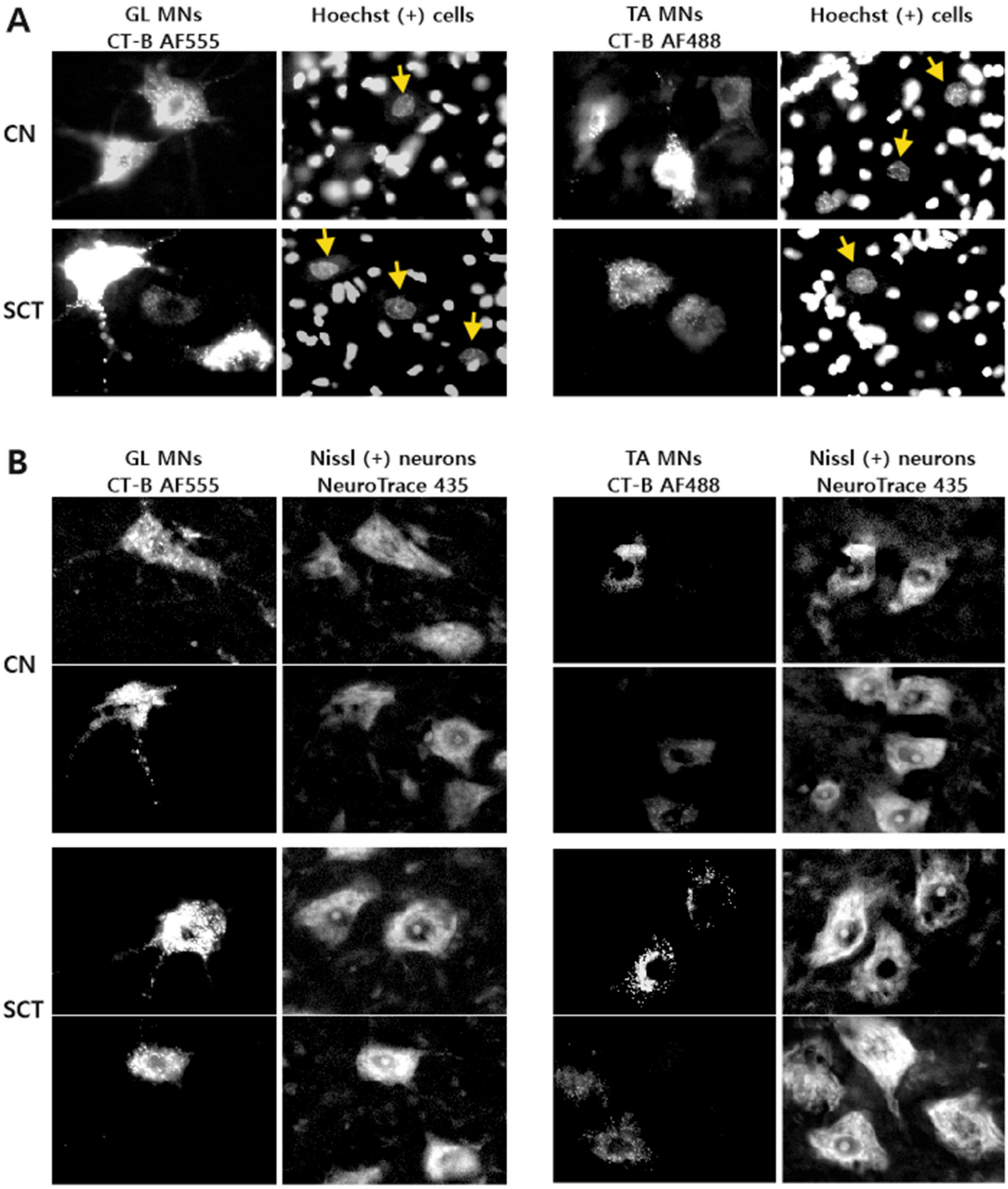

Figure S1. Hoechst and Nissl stain of GL and TA MNs. Hoechst 33342 staining (**A**) and NeuroTrace 435/455 blue fluorescent Nissl stain (N21479, Invitrogen, Waltham, MA, US) (**B**) were conducted to identify the physiological state of the MNs on the spinal cord transverse sections containing GL and TA MNs from CN and SCT animals. Neither chromatin condensation nor apoptotic profiles were detected (nuclei of the traced MNs are marked with yellow arrow in (**A**) and the distribution of the Nissl substance in the MNs are comparable between CN and SCT animals (**B**). MN—motoneuron; GL—gastrocnemius lateralis; TA—tibialis anterior; CN—control; SCT—complete spinal cord transection; CT-B AF - Cholera Toxin Subunit B conjugated with Alexa Fluor dye.

The free-floating sections were rehydrated for 40 minutes in 0.1 M phosphate-buffered saline (PBS), pH 7.2. Then the sections were washed for 10 minutes in PBS plus 0.1% Triton® X-100 to permeabilize the tissue. After wash (two times for 5 minutes each) in PBS, the sections were incubated either with Hoechst solution (diluted in PBS 1:500) for 5 minutes or with NeuroTrace stain solution (diluted in PBS 1:50) for 20 minutes. Next, the sections were washed in PBS plus 0.1% Triton X-100 for 10 minutes and three times (5 minutes each) in PBS. Sections were mounted on slides with mounting medium ProLong™ Diamond Antifade Mountant (P36970, Invitrogen, Waltham, MA, US).

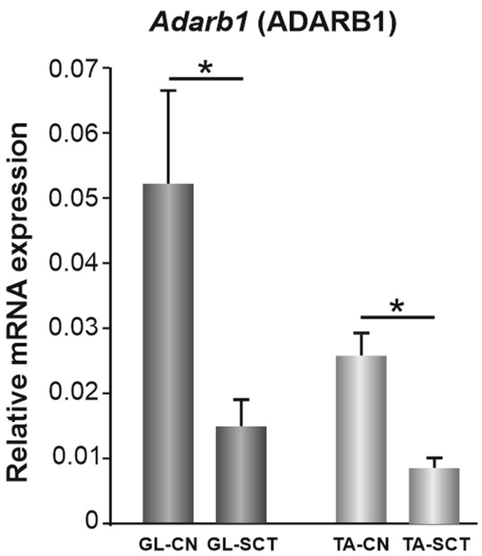

Figure S2. SCT downregulated the transcript level of *Adarb1* gene, coding for ADAR2 enzyme. N=9 both for CN and SCT group; Data are means +/− SEM; Mann-Whitney U-test, * $p < 0.05$.

Table S1. List of probes and primers.

Gene Symbol	Name of Coded Protein	Accession Number	Roche UPL No.	Forward Primer (5′–3′)	Reverse Primer (5′–3′)	Amplicon Length/nt
Gria1	AMPAR GluA1	NM_031608.1	69	tcccttgaccataaccttgg	actcatgaatggcttggagaa	61
Gria2	AMPAR GluA2	NM_017261.2; NM_001083811.1	67	ggaggtgattccaaggaaaag	cccccgacaaggatgtaga	77
Gria3	AMPAR GluA3	NM_032990.2; NM_001112742.1	94	ccaagaatgcaccactgaag	ctgcgggagacatccact	109
Gria4	AMPAR GluA4	NM_017263.2; NM_001113184.1; NM_001113185.1	25	ggctaccattatatcatcgcaaa	ggtgtattaaaatctaccaactggaat	110
Grin1	NMDAR GluN1	NM_001270602.1	95	cctacacagctggcttctacag	cgaaggaaactcaggtggat	94
Grin2a	NMDAR GluN2A	NM_012573.3	94	cgtcatggtctccaggagtaa	cactgaagggttcgaggaaa	62
Grin2b	NMDAR GluN2B	NM_012574.1	106	tcctgcagctgtttggagat	gctgctcatcacctcattctt	95
Grin2c	NMDAR GluN2c	NM_012575.3	94	ggcactcctgcaacttctg	gttctggcagatccctgaga	76
Grin2d	NMDAR GluN2d	NM_022797.1	25	acatggtgcgatacaaccag	tgaaggcgtccagtttcc	75
Gabra1	$GABA_AR$ α1	NM_183326.2	129	tgacagtcattctctcccaagtc	tcagaacggtcgtcactcc	87
Gabra2	$GABA_AR$ α2	NM_001135779.2	94	gacagacttctggatggttacga	ggtcacatagatgttggtggag	87
Gabra3	$GABA_AR$ α3	NM_017069.3	129	cctacttgccatgtatcatgactg	ggtcatggtgagaacagtggt	113
Gabrb3	$GABA_AR$ β3	NM_017065.1	80	atcgagctcccacagttctc	tcaatgagagtcgagggtagg	91
Gabrg2	$GABA_AR$ γ2	NM_183327.1	75	ttgaagtgggagacacaaggt	cggacataaccacatagtcacc	111
Glra1	GlyR α1	NM_013133.1	69	ccggcaacacaaggaact	tctccaccctcatcatcctt	69
Glrb	GlyR β	NM_053296.1	29	ggatctattcaagagacaacaatg g	gcttgagtctggggtcgtt	79
Slc12a4	KCC1	NM_019229.2	101	acagagcgggatcgagag	ctctggtccatgtcatctgg	130
Slc12a5	KCC2	NM_134363.1	75	ttctggacaaccaccctca	ggcagaagcccttcacct	84
Slc12a6	KCC3	NM_001109630.1	124	gcgtcttacctgggtagtgg	tggagatggcagttaacattgta	95
Scn1a	Nav1.1	NM_030875.2	67	agaaccaggccacattggag	gttctgatgctgttgctgcc	120
Scn3a	Nav1.3	NM_013119.2	80	acagcgagagcaggagaga	cgtttcagtggtggtaccgt	83
Scn8a	Nav1.6	NM_019266.3	67	agagcaaaaagaggccgagt	tcttcaatggcgtcttccga	120
Scn9a	Nav1.7	NM_133289.2	129	ggtcatggtgattgggaacc	ttgtttgcatcggtgtcttcc	111
Cacna1b	Cav2.2	NM_001195199.1 NM_147141.1	73	cggcactggagatcaagc	gcagattagcccacacagaag	96
Cacna1d	Cav1.3	NM_001389225.2	69	cccttatccaccctgcgatg	agattcttcgaggacgggga	98

Kcnn1	SK1 (K_{Ca}2.1)	NM_019313.1	124	tcggaaacaccagcgtaagt	cttcacagtccggagcttct	64
Kcnn2	SK2 (K_{Ca}2.2)	NM_001309404.1; NM_019314.2	113	gcgttttgttatgaagactttaatga	gtgacatcctgttgatcatggta	132
Kcnn3	SK3 (K_{Ca}2.3)	NM_019315.2	69	ttcagggctcccaattca	atttagctggctgccttgc	72
Kcnma1	KCa1.1	NM_031828.1	56	cctcacacagccctttgc	gggtgaggatattgtcattgaag	95
Grm1a	mGluR1A	NM_017011.1	101	caccgaagaagatgaattgga	gaatcctcaggggtcagctt	69
Grm5	mGluR5	NM_017012.1	82	tccagcagcctagtcaacct	gattttccgttggagcttagg	71
Htr1a	5-HTR1A	NM_012585.1	113	gggcaactccaaagagcac	cgggggcataggagttagat	62
Htr2a	5-HTR2A	NM_017254.1	69	agcactcgagccaaactagc	tggaagagcttttctgatgaca	71
Htr2b	5-HTR2B	NM_017250.2	129	cgcggtaataatccccacca	ccaccgccaaggacattaga	110
Htr2c	5-HTR2C	NM_012765.3	69	aagaaggtgttcgttggcct	ccgtttctcgtctagctgcc	106
Htr3a	5-HTR3A	NM_024394.2	65	ggctaactacaagaagggagtgc	accatatgtaggtggtcagaacc	131
Adra1a	NAα1A	NM_017191.2	50	gcgtctgggtgctttctttg	ggctcctcattgatctggca	100
Adra1b	NAα1B	NM_016991.2	129	ttggctccccttcttcatcg	gagcacgggtagatgatggg	135
Adra1d	NAα1D	NM_024483.2	129	tgtgctggttccccttcttc	agcgggttcacacagctatt	124
Adarb1	ADARB1 (ADAR2)	NM_012894.2	64	aagatcaccaagcccaccac	gaaggcagtgaacagacgtg	87
GAPDH	GAPDH	NM_017008.4	*	ctgcaccaccaactgcttag	tgatggcatggactgtgg	92

* sequence: tttggcatcgtg. Service provided by DNA Sequencing and Synthesis Facility, Institute of Biochemistry and Biophysics, Polish Academy of Sciences, Warsaw, Poland.
